# Evaluation of *Bacillus coagulans* LMG S-31876 for immunomodulation and stress: a double-blind, placebo-controlled clinical trial

**DOI:** 10.3389/fnut.2024.1484499

**Published:** 2025-01-15

**Authors:** Ranjith Kumar Kallur, Sreenadh Madapati, Mayuri Banerjee, Ankita Mathur, Sourish Bhattacharya

**Affiliations:** ^1^Abode Biotec India Pvt. Ltd., Hyderabad, Telangana, India; ^2^Process Design and Engineering Cell, CSIR-Central Salt and Marine Chemicals Research Institute, Bhavnagar, Gujarat, India

**Keywords:** ProBC Plus (*Bacillus coagulans* LMG S-31876), immune system, stress, clinical trials, health, placebo

## Abstract

**Objective:**

The study aimed to analyze the safety and effectiveness of the ProBC Plus (*Bacillus coagulans* LMG S-31876) supplement across various health parameters, including stress levels, immunoglobulin levels, biochemical parameters, and vital signs.

**Methods:**

A randomized, double-blind, placebo-controlled clinical trial study was conducted involving 50 subjects diagnosed with ailments related to immune system dysfunction and stress related disorders. Patients were treated with ProBC Plus (2 billion colony-forming units [CFU]) along with a placebo capsule administered once daily for a period of 8 weeks.

**Results:**

The effects of ProBC Plus exhibited a positive response on stress relief, lipid parameters, immune status, and vital signs, which is further statistically significant (*p* value <0.05, 5% marginal error at 95% CI).

**Conclusion:**

The study on ProBC Plus showed positive results. Over the course of 8 weeks, an improvement in the immune status was observed, as indicated by the immune status questionnaire. Enzymatic markers exhibited a significant decline in predicting a positive response toward treatment. In terms of lipid profile, ProBC Plus helps to maintain the value within the normal range, thereby predicting its potential as cardiovascular support. The vital signs remained within the normal range throughout the study. Therefore, ProBC Plus is considered safe for consumption and contributes to the overall well-being of individuals.

**Clinical trial registration:**

https://ctri.nic.in/Clinicaltrials/pmaindet2.php?trialid=77914&EncHid=24313.96864&userName=CTRI/2023/01/048720, CTRI/2023/01/048720.

## 1 Introduction

The immune system consists of complex interactions between cells and tissues that work together to protect the body against infections and diseases (1, 2). The intricate relationship between the immune system and overall well-being has emerged as a significant area of focus in healthcare (3). Concurrently, the impact of stress on the processes (physiological) has garnered attention because of the disruption of the body (4). Among these existing scientific endeavors, immunomodulation has gained quite prominence. Immunomodulation involves modifying immune responses, potentially enhancing disease resistance (5), and neutralizing the negative effects of chronic stress (6, 7).

Immunomodulation is often defined as the deliberate regulation of immune responses that has profound implications for human health (8–12). Immune system signaling molecules play a pivotal role in eliminating abnormal cells, and maintaining homeostasis. The ability to modulate these responses opens up new therapeutic options that extend beyond the traditional treatment. The impact of successful immunomodulation extends to wide applications such as (a) chronic disease management, (b) cancer immunotherapy, (c) stress resilience, and (d) infectious disease management (13). The immune system and stress are interconnected, and the interplay between psychological and physiological states plays an important role in balancing these two factors. Numerous factors influence an individual’s resistance to infections such as physical activity, stress, age and smoking, alcohol consumption, and medications (7). Despite the availability of many other treatment options, probiotics are considered as a prominent choice in recent times (4). Probiotics are currently defined as live microorganisms that, when administered in adequate amounts, provide health benefits to the host. This definition reflects extensive research demonstrating that probiotics can enhance host health and treat various infectious and non-infectious pathologies (14, 15).

*Bacillus coagulans* is a potent organism and has been identified within the realm of nutrition and health. Its clinical evidence has established improvements in gastrointestinal tract (GIT) and immune system regulation (15). Subsequently*, B. coagulans* as a probiotic has garnered attention because of its most important ability to withstand the harsh conditions in the gut environment and provide beneficial effects on immune function (16).

*Bacillus coagulans* LMG S-31876, which has been isolated from fermented rice and known for spore-forming, exhibits the ability to withstand harsh conditions to acidic and gastric juices. The strain is well characterized with accession number MZ687045. It has been deposited with reference number LMG S31876 in BCCM/LMG and MTCC 25396 in MTCC-IDA. The whole genome sequence of *B. coagulans* LMG S-31876 is available in the DDBJ/EMBL/GenBank database under the accession number JANKOH000000000. The strain is safely deposited with the reference code ProBC Plus (17).

In the present scenario, the bridge between immunomodulation and stress remains an area for investigation. While there is enormous research that provides insights into *B. coagulans* as probiotics and stress on immune function, few studies have delved into immune-modulating properties, addressing the interplay between stress, immune responses, and their interventions. Some have assessed the influence under stress conditions (18, 19). There is a scarcity of well-designed clinical studies that evaluate the potent application of *B. coagulans* which regulates immunomodulation and stress levels. This research addresses a gap in understanding the potential of *B. coagulans* in clinical trials that utilize randomized, double-blind, and placebo-controlled methodologies. Henceforth, these studies aim to enhance our knowledge and examine how *B. coagulans* may help mitigate stress-induced immune changes. In light of this research gap, the administration of a *Bacillus coagulans* (LMG S-31876)-based product, ProBC Plus, is expected to yield measurable improvements in immunomodulation and stress levels compared to a placebo.

## 2 Materials and methods

### 2.1 Research design

#### 2.1.1 Study design and duration of the study

The study is a double-blind, randomized, placebo-controlled clinical trial that evaluates the efficacy and safety of the investigational product *Bacillus coagulans* LMG S-31876 (ProBC Plus) (10^6^–10^8^ colony-forming units [CFU]) in the treatment of immunomodulation and stress levels. A total of 50 subjects are included, and the duration of the study is up to 8 weeks. For many gastrointestinal disorders (e.g., IBS, constipation, and diarrhea), clinical effects may take time to manifest. 8 weeks is a reasonable duration for observing changes in symptom relief, gut motility, and inflammatory markers. Furthermore, to monitor the potential adverse effects of probiotics, such as bloating, gas, or infection (especially in immunocompromised patients), an 8-week trial duration provides sufficient time to assess both common and rare side effects while balancing the risk of long-term harm.

**Figure 1:**
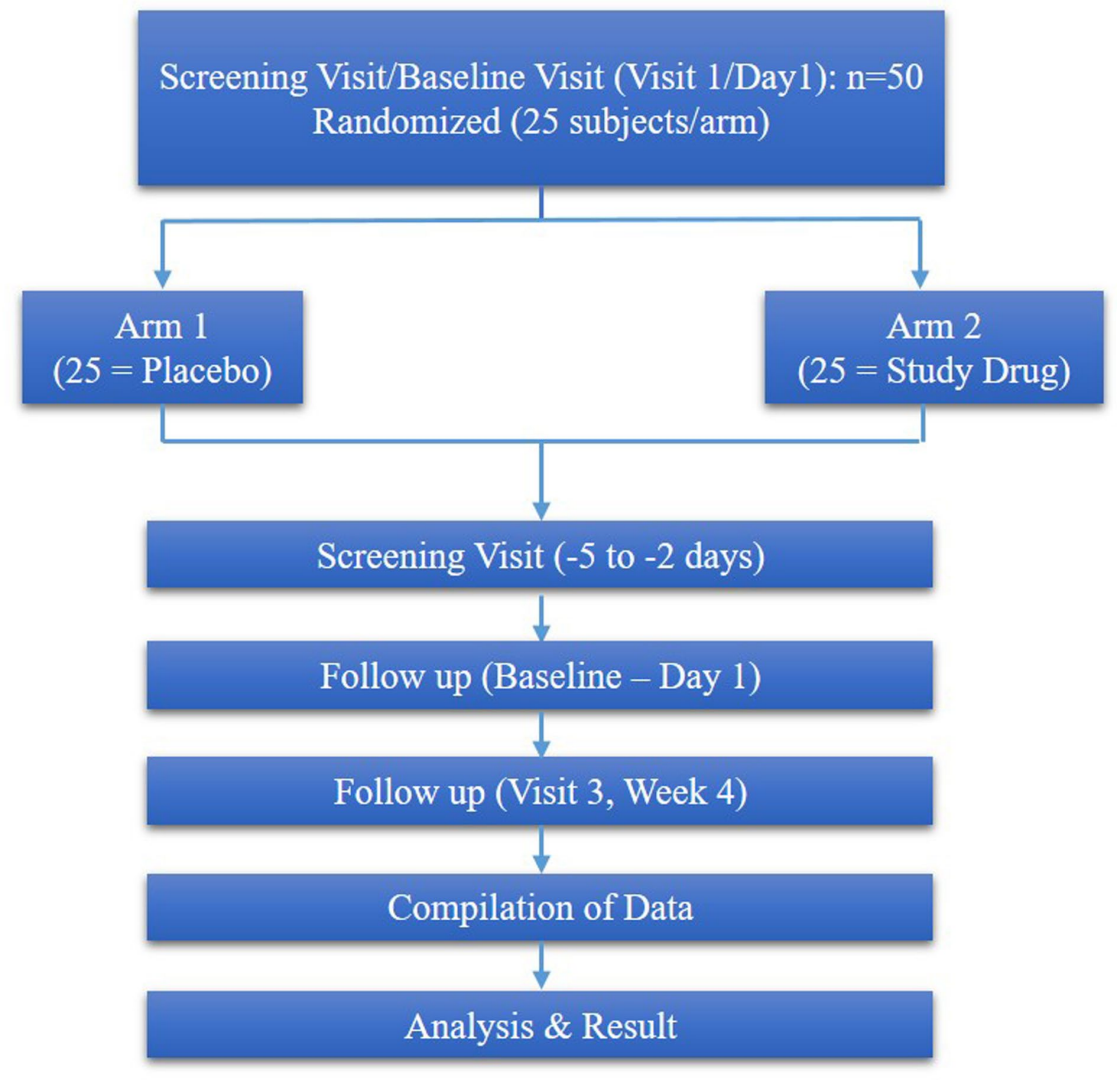
Consort flow diagram.

The study consisted of 4 visits, such as screening visits (−5 to −2 days), baseline (visit 1) and week 4 (visit 4), week 8 (visit 8) respectively (Figure 1).

### 2.2 Selection of participants and demographic variables

A total of 50 healthy individuals, including 18 male individuals and 32 female individuals from the Asian population, were selected for the study. All the participants aged between 18 and 60 years. Demographic variables, including age, sex, and medical history (encompassing past and current co-morbidities) were documented. Table 1 shows the summary of demographics (ITT population). The mean age of the ProBC Plus treatment group and the placebo groups are comparable. The standard deviation (SD) indicates the degree of dispersion around the mean. A symmetrical distribution is suggested by similar mean and median values.

**Table 1:** Represents the summary of demographic characteristics (ITT population).

Parameters	Statistics	ProBC plus	Placebo
		(*N* = 25)	(*N* = 25)
Age (Yrs)	*n*	25	25
	Mean	37.72	37.88
	SD	12.17	10.43
	Median	37	37
	Min, Max	1,960.00	1,858.00
Weight (Kg)	*n*	25	25
	Mean	63.83	65.42
	SD	7.14	10.4
	Median	64.4	68
	Min, Max	4,876.20	3,782.00
Height (cm)	*n*	25	25
	Mean	163.6	161.6
	SD	7.05	5.16
	Median	162	159
	Min, Max	1,52,180.00	1,51,171.00
Gender			
Female	*n* (%)	17 (68.0)	15 (60.0)
Male	*n* (%)	8 (32.0)	10 (40.0)
Race			
Asian	*n* (%)	25 (100.0)	25 (100.0)

The mean weight of the ProBC Plus group is marginally lower that of the placebo group. Greater variability is shown by the relatively high SD in the placebo group. The weight distribution is comprehensively outlined by the mean and median. Similarly, regarding height, the ProBC Plus group has a slightly higher mean height. The higher SD in the ProBC Plus group suggests greater variability.

There are a higher proportion of female participants in the research treatment and placebo groups as compared to male participants. Assessing population diversity is aided by the clear portrayal of gender distribution.

#### 2.2.1 Criteria for the selection of the participants

The subjects were selected based on the following inclusion and exclusion criteria.

1. Inclusion criteria: (a) fasting blood glucose (≤110 mg/dL) (b) normal blood parameters (Hb ≥ 10 g/dL) (c) voluntary written informed consent to participate in the study (d) a clear understanding of the nature and purpose of the study with the potential risks and side effects.
2. Exclusion criteria: (a) participants undergoing antibiotic treatment (b) participants suffering from gastrointestinal diseases, diabetes, and chronic immunodeficiency, abnormal blood pressure, allergic to any ingredients in the study product (c) participants who smoke or consume alcohol (d) participants who have participated in the trials over the last 3 months (e) pregnant or lactating women.

### 2.3 Treatment and study protocol

The study included two groups. Group I received the probiotic *B. coagulans* LMG S-31876, (10^6^–10^8^ CFU) (2 billion CFU/capsule/day) (Investigational product-IP), and Group II received a placebo. The reference treatment is a placebo capsule that contains starch in equivalent weight per capsule as that of IPs.

### 2.4 Assessment of treatment efficacy and safety

The primary outcome of the study includes the stimulatory effect of Pro BC Plus on the immune system. This is measured using Immune Status questionnaire (ISQ) and Perceived Stress Scale (PSS) scores between the intervention and placebo groups over a period of 8 weeks. Secondary outcomes include the differences in the (a) hematological parameters and (b) immune parameters between the intervention and placebo groups over the same 8-week timeframe.

### 2.5 Assessment of study tools

PSS (Perceived Stress Scale): This scale is utilized to measure stress levels. It consists of ten questions designed to evaluate individual life stress. Individual scores on the PSS can range from 0 to 40 with the higher scores indicating higher perceived stress, 0–13 denoting low stress, and 14–26 indicating moderate stress. The difference in the PSS score was analyzed between the intervention and placebo groups over the study period.

ISQ (Immune Status Questionnaire): This questionnaire consists of seven immune-associated symptoms that are scored on a five-point Likert scale ranging from 0 to 4. Changes in the ISQ score from baseline to 8 weeks of treatment were analyzed between the intervention and placebo groups.

Analysis of vital parameters: The parameters such as pulse rate, systolic blood pressure, and diastolic blood pressure, fasting blood sugar (FBS) were assessed from visit 1 to visit 4.

Analysis of enzymatic activity: Enzymes such as alanine transaminase (ALT/SGPT), alkaline phosphatase (ALP), and aspartate aminotransferase (AST) were analyzed during visits 1 and 4 for groups I and II.

Immunoglobulin activity: Immunoglobulins, such as IgG, IgA, and IgM, were analyzed during visits 1 and 4 for groups I and II.

Ethical considerations and clinical trial registration: The study at the investigational site, protocol, subject information sheet (SIS), and all related documentation were submitted to the Institutional Ethics Committee (IEC). The clinical trial was registered in the CTRI under the number CTRI/2023/01/048720. https://ctri.nic.in/Clinicaltrials/pmaindet2.php?trialid=77914&EncHid=24313.96864&userName=CTRI/2023/01/048720.

## 3 Statistical significance

The *p*-values for the ISQ and PSS were calculated using an analysis of covariance (ANCOVA) model, which measures changes from baseline. Additionally, statistical significance was observed over time along with confidence intervals (CIs). Comparisons between ProBC Plus and placebo at both week 4 and week 8 were based on this ANCOVA analysis. Variations in hematology, biochemistry, and immunoglobulin parameters from baseline to the end of treatment are assessed using a two-sample t-test with GraphPad version 7. Furthermore, all comparisons between groups for parameters such as ALT, ALP, AST, and immunoglobulin levels were performed using the two-sample t-test.

## 4 Results

### 4.1 Assessment of immune status questionnaire

**Figure 2:**
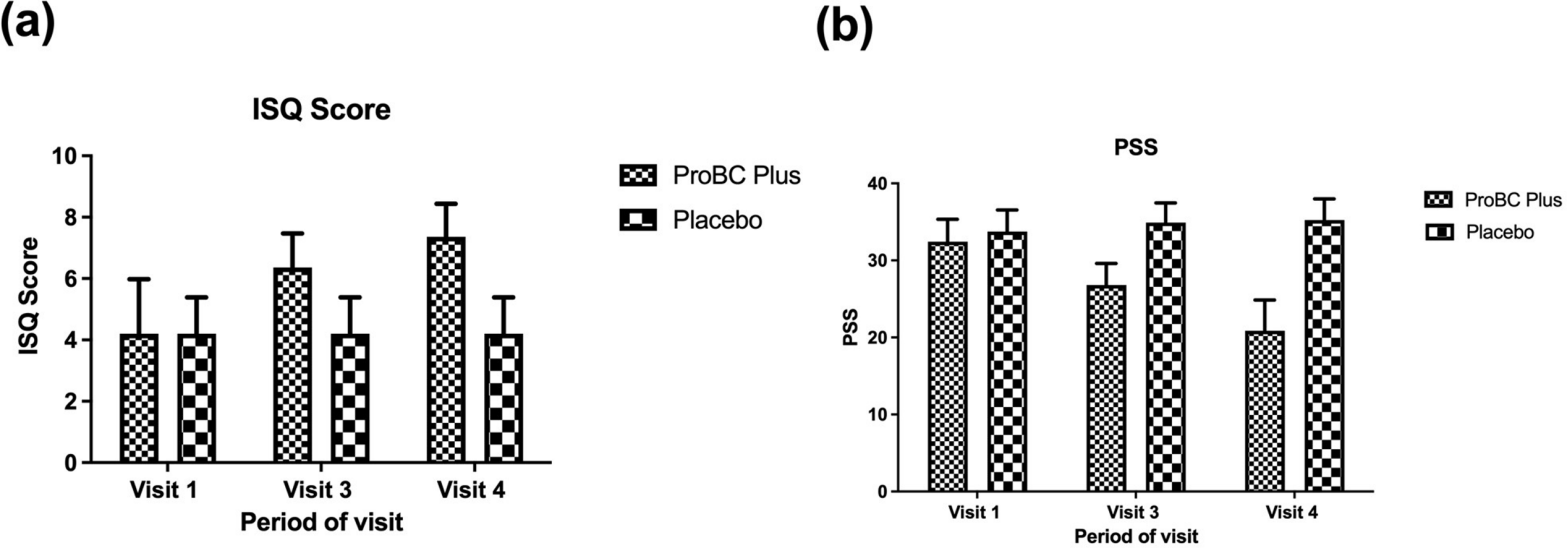
**(A)** Estimation of changes in ISQ (Immune Status Questionaire) Score across visit 1 to 4. **(B)** Estimation of changes in PSS (Perceived Stress Scale) across visit 1 to 4.

Initially, at baseline, there were no significant differences between the placebo and ProBC Plus. However, by week 4, the mean change from baseline for ProBC Plus was greater than that of placebo, with a *p*-value of less than 0.0001, suggesting statistical significance. A continuous effect of ProBC Plus was indicated through consistency in statistical significance. The strong impact of ProBC Plus was demonstrated by statistically significant results observed across multiple time points. Improvement in immune status was more evident in weeks 4 and 8. Over time, the study product, ProBC Plus, showed a favorable change in immune status when compared to the placebo, achieving statistical significance (*p* < 0.0001). Furthermore, the statistical analysis, conducted using an ANCOVA model, revealed that the observed changes were both statistically significant and clinically relevant, with the ProBC Plus group achieving higher mean ISQ scores and narrower confidence intervals compared to the placebo group. A progressive improvement over time underscores the efficacy of ProBC Plus in enhancing immune status. These findings, supported by a highly significant *p*-value (*p* < 0.0001), suggest that ProBC Plus had a substantial and beneficial impact on the immune modulation of the participants throughout the study period. This indicates that the study product significantly influenced the immune status of the participants, as shown in Figure 2.

### 4.2 Analysis of perceived stress scale

At baseline, there was no statistically significant difference between the study product and the placebo. However, by week 4, the study product showed a reduction in perceived stress levels compared to the placebo, achieving a statistically significant difference (*p* < 0.0001). By week 8, the ProBC Plus group exhibited a substantial decrease in PSS scores from baseline compared to the placebo. Therefore, the study product resulted in a reduction of PSS levels among the participants. The analysis shows that the study product is effective in mitigating the stress (perceived) and can lead to the overall well-being of the individuals. Moreover, the statistical significance (*p* < 0.0001) clearly demonstrates the application of the developed product in reducing perceived stress over time. The observed decrease in PSS scores, coupled with the low variability (as indicated by narrow confidence intervals), supports the conclusion that ProBC Plus plays a significant role in stress reduction. Thus, there is a clear connection between the use of this product and reduced stress levels.

### 4.3 Determination of biochemical activities

**Figure 3:**
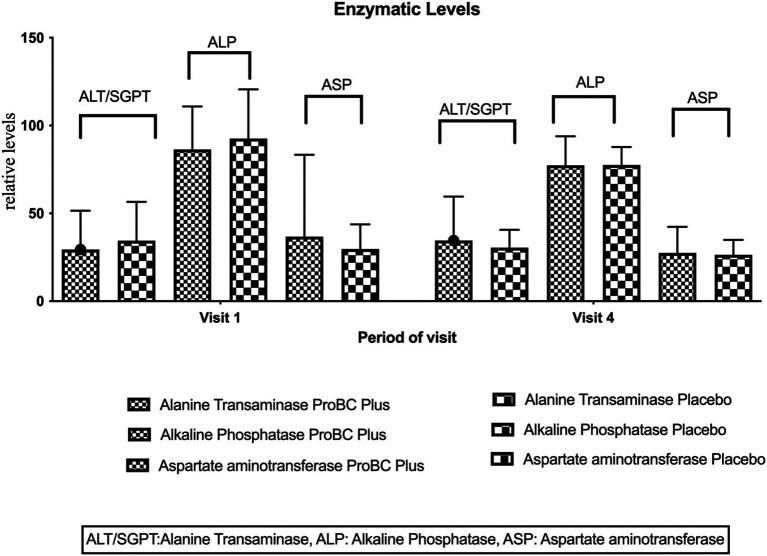
Represents the comparative enzymatic levels between the ProBC Plus and placebo.

Alanine Transaminase (ALT/SGPT): The mean ALT levels at baseline and week 8 was found to be statistically insignificant between the study product and the placebo. The baseline comparison (*p*-value [1] = 0.4286) suggests no statistically significant difference in ALT/SGPT levels between the ProBC Plus and placebo groups at the start of the study. Similarly, the paired test (p-value [2] = 0.4940) for visit 4 indicates no statistically significant difference in ALT/SGPT levels between the study product and placebo groups. These findings suggest that ProBC Plus did not have a significantly impact on ALT levels compared to the placebo. Additionally, there was considerable variability in ALT levels throughout the trial period, as illustrated in Figure 3, which may have contributed to the lack of significant differences observed.

Alkaline Phosphatase (ALP): The ALP levels at baseline and week 8 remained stable, showing no significant increase or decrease. At baseline, the study product recorded higher ALP levels compared to the placebo. This trend continued at week 8, indicating stable ALP levels over the study period. The baseline comparison (*p*-value = 0.4026) suggests no statistically significant difference in ALP levels between the study product and placebo groups. This finding suggests that ProBC Plus had a significant impact on ALP levels during the study period, showing a notable reduction compared to the placebo group.

These findings indicate that, although baseline ALP levels were similar between the two groups, ProBC Plus may help reduce ALP levels over time, supporting its potential as an effective intervention.

Aspartate aminotransferase (AST): AST levels exhibited a slight decline from baseline to week 8, with a slight variability observed between the study product and placebo. This suggests a favorable response to the treatment being investigated, signifying the beneficial effect on AST levels within the study population. The baseline comparison (*p*-value = 0.4760) suggests no statistically significant difference in AST levels between the study product and placebo groups at the start of the study. The paired t-test (*p*-value = 0.3733) for Visit 4 indicates no statistically significant difference in the changes in AST levels between the study product and placebo groups. These results suggest that, although there was a slight reduction in AST levels, the effect of ProBC Plus on AST was not substantial enough to achieve statistical significance within the duration of the study.

### 4.4 Estimation of hematology parameters

**Figure 4:**
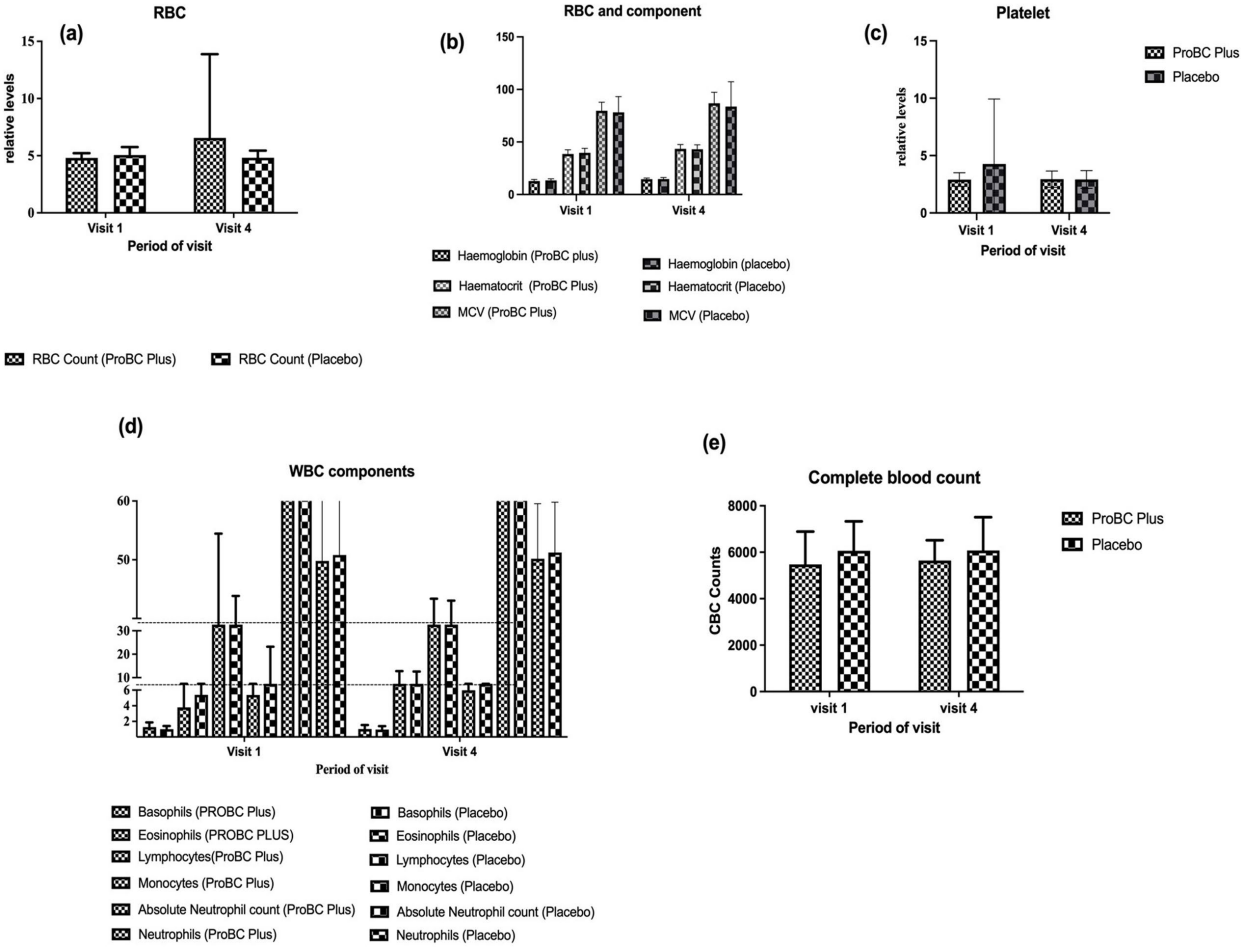
Depiction of hematology parameters between ProBC Plus and placebo **(A)** RBC **(B)** Hemoglobin, Hematocrit, and MCV (Mean Corpuscular Volume) **(C)** Platelet count **(D)** WBC components including basophils, eosinophils, lymphocytes, absolute neutrophil count and neutrophils **(E)** Complete blood count.

Hematology parameters were found to be in the normal range. The RBC count shows an increase in the number of RBC cells per unit volume by visit 4 as compared to the placebo (Figure 4A). However, the difference was not statistically significant. (b) The components of RBC, including hemoglobin, hematocrit, and MCV (mean corpuscular volume), were found to be in the normal range and did not show any increase over time. (c) Platelet count remained in the normal range throughout the study (Figure 4B) (d) The components of WBC, including basophils and eosinophils, were in the normal range and did not change significantly. Although the lymphocyte count was found to be statistically insignificant over time, the mean value decreased with time. The mean neutrophil count exhibited a slight increase, while the absolute neutrophil count declined over the study period (e) Additionally, the complete blood count exhibited almost similar levels as compared to that observed in the placebo group.

### 4.5 Analysis of lipid profile activity

**Figure 5:**
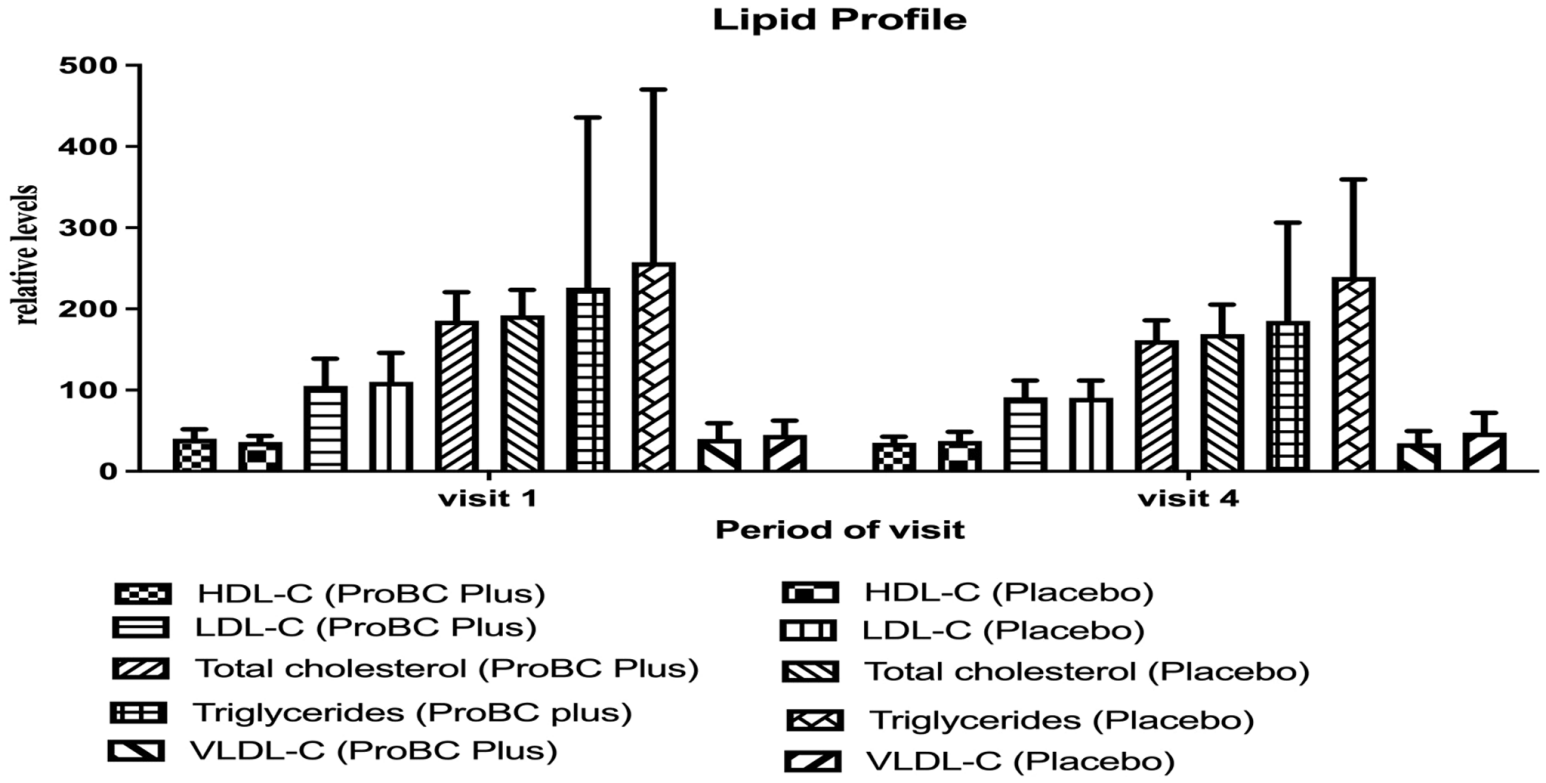
Estimation of lipid profile activity (HDL-High Density Lipoprotein, LDL-Low Density Lipoprotein, Total Cholesterol and Triglycerides, VLDL-Very Low-Density Lipoproteins).

In the case of mean high-density lipoprotein (HDL-C), low-density lipoprotein (LDL-C), total cholesterol, very low-density lipoprotein (VLDL), and triglycerides exhibited a similar decline in values during the period of time. The values were within the normal range (Figure 5).

### 4.6 Estimation of serum immunoglobulin levels

**Figure 6:**
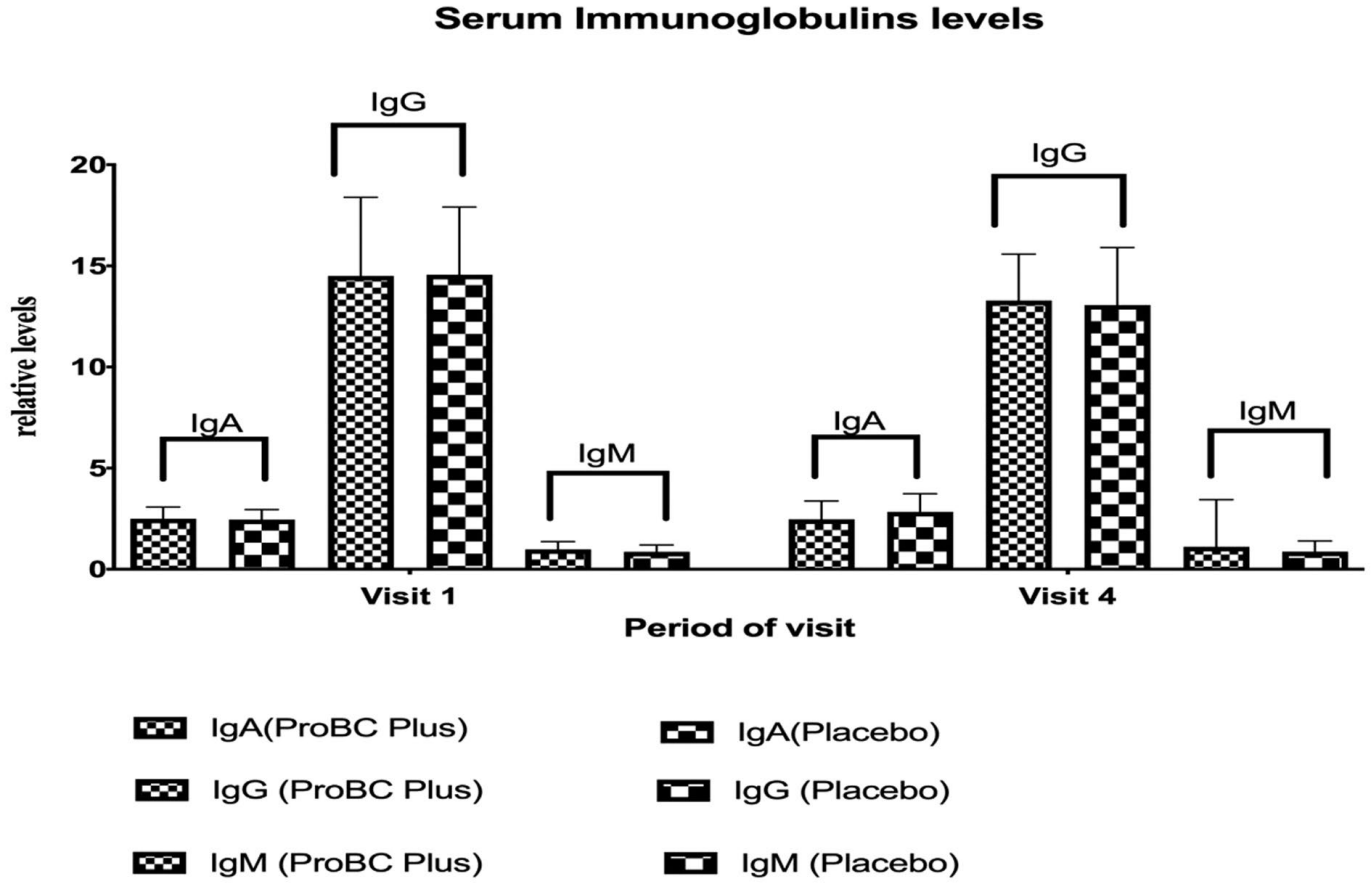
Estimation of serum immunoglobulins (IgA, IgG and IgM).

There were no statistically significant differences in IgA levels between the ProBC Plus and placebo groups at baseline (Visit 1) or Week 8 (Visit 4) assessments. Similarly, there were no statistically significant differences in IgG and IgM levels (Figure 6) between the ProBC Plus and placebo groups at baseline (Visit 1) or Week 8 (Visit 4) assessments. Both groups showed variability in IgG levels throughout the trial, but these changes were not statistically significant.

### 4.7 Estimation of vital signs

**Figure 7:**
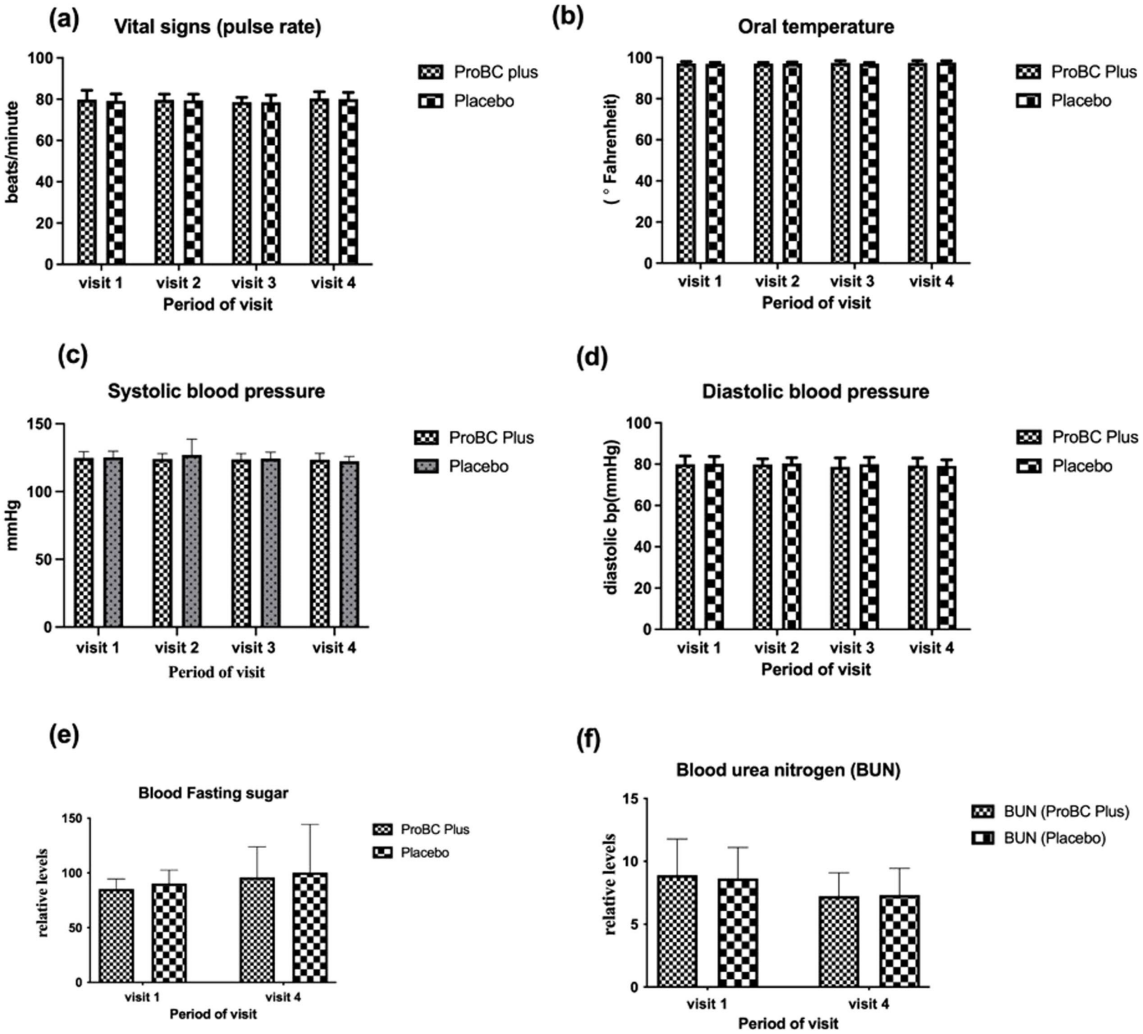
Depiction of vital parameters **(A)** Pulse rate **(B)** Oral temperature **(C)** Systolic blood pressure **(D)** Diastolic blood pressure **(E)** Blood fasting sugar **(F)** Blood urea nitrogen (BUN).

The vital signs such as pulse rate, oral temperature, systolic and diastolic blood pressure, blood fasting sugar, and blood urea nitrogen (BUN levels) were all found to be within the normal range, and these parameters were found to be significant at baseline (Figure 7). It was noted that the error bars were large, possibly due to the impact of sample size. Smaller sample sizes may result in larger error bars, making it difficult to detect significant differences. However, with the exception of RBC and platelets, all other measurements had smaller error bars.

## 5 Discussion

The beneficial effects of probiotics are very well-known for many years (20). Several studies have provided insights into *Bacillus coagulans*, highlighting it as a potent probiotic that supports immunomodulation. According to a study, *Bacillus coagulans* GBI-30, 6,086 provides a positive response to the immune system (16). The capacity of probiotics to affect both innate and adaptive immunity facilitates immunomodulation as an important consideration when choosing a probiotic strain. The activities of pro- and anti-inflammatory cytokines that regulate T and B cells boost the immune system’s defense against infections. Subsequently, probiotics assist in the reduction of inflammation and provide a shield to the system. Additionally, they strengthen the intestinal barrier and enhance immunological responses, including the activity of natural killer cells and macrophages, which enhances resistance to infections. These characteristics are important for identifying probiotic strains with promising medicinal applications (21, 22). Previous reports suggest that spore may have immunomodulatory effects and can stimulate a cascade of events in response to cellular activities (23–25). In a previous study, *Bacillus coagulans* MTCC 5856 exhibited a substantial immune response, and the spores exhibited maximum survival (92%) (26).

Amid extensive research on *Bacillus coagulans* as a probiotic and its immunomodulatory properties, the current study deals with the effects of ProBC Plus compared to a placebo on various health parameters. ProBC Plus exhibited a notable improvement in immune status from baseline to week 8, as determined through an immune status questionnaire. Subsequently, while analyzing the perceived stress scale, the reduction in stress over a period of time signifies a promising result. According to some of the studies reported (27, 28) chronic stress can affect the GIT and weaken the nervous system. Therefore, the ability of ProBC Plus to mitigate stress may contribute positively to the overall well-being of an individual. In addition, ProBC Plus exhibited better results in lipid profiles, with values remaining within the normal range. Hence, ProBC Plus may prove as a potent cardiovascular product. A comparable outcome (29, 30) was observed in the reduction of cholesterol levels with *B. coagulans* MTCC 5856. Similarly, in this study, the enzymatic levels of alanine transaminase, alkaline phosphatase, and aspartate aminotransferase were found to be within the normal range.

Additionally, the levels of immunoglobulins, such as IgA and IgG, showed a decline over the study period, while IgM indicated a slight increase. Although the results were not statistically significant, this may be due to the study population and the diversity of the population in terms of age, genetics, and overall health. Vital signs remained within the normal range throughout the study and did not vary from baseline. This indicates that ProBC plus did not adversely affect the vital parameters. As a positive outcome, the study product ProBC Plus is safe for consumption and did not alter any physiological processes.

## 6 Conclusion

The study demonstrated multitudinous positive outcomes related to the utilization of ProBC plus, a product containing *Bacillus coagulans* LMG S-31876. (a) Immunoglobulin activity: changes in immunoglobulin levels (IgA, IgG, IgM) were observed, although they were not statistically significant. The diversity of the study population may have contributed to variability in results. (b) Immune status and stress reliever: ProBC Plus exhibited a notable improvement in participants’ immune status over the 8-week period, as evidenced by the immune status questionnaire. The clinical study on probiotics as an immunomodulator presents promising results in terms of ISQ and PSS scores, with improvements and stability in vital signs. (c) Enzymatic Levels: Alkaline phosphatase and aspartate aminotransferase exhibited a significant decline, providing a promising response to the treatment (d) Lipid profile: ProBC Plus assisted in maintaining lipid values within a normal range, suggesting its potential as a cardiovascular support product (e) Vital measurement: The parameters remained within the normal range throughout the study, indicating that ProBC Plus did not adversely affect physiological processes. This suggests that the product is safe for consumption and will aid in overall health and well-being.

### Probiotic strain details

Pro BC plus *Bacillus coagulans* MTCC25396 (India).

Pro BC plus *Bacillus coagulans* SD-7789 ATCC [USA].

Pro BC plus *Bacillus coagulans* LMG S-31876 BCCM LMG [Belgium].

## Data Availability

The original contributions presented in the study are included in the article/supplementary material, further inquiries can be directed to the corresponding authors.
